# Dexmedetomidine plus sufentanil for pediatric flexible bronchoscopy: A retrospective clinical trial

**DOI:** 10.18632/oncotarget.17169

**Published:** 2017-04-17

**Authors:** Xiujing Dang, Weidong Hu, Zhendong Yang, Shiyu Su

**Affiliations:** ^1^ Department of Anesthesiology, Qilu Children's Hospital of Shandong University, Jinan, Shandong, 250022, P.R. China; ^2^ Department of Anesthesiology, The Fifth People's Hospital of Jinan, Jinan, Shandong, 250022, P.R. China

**Keywords:** dexmedetomidine, sufentanil, pediatric, flexible bronchoscopy

## Abstract

Several studies have reported the use of dexmedetomidine (DEX) plus opioids for flexible bronchoscopy in both adults and children. To determine whether DEX plus sufentanil (SF) is safe for children, 142 children undergoing flexible bronchoscopy were assigned to one of three groups, each of which received the same SF loading dose and similar DEX and SF maintenance doses, but different loading doses of DEX: DS1 (DEX 0.5 μg·kg^–1^), DS2 (DEX 1.0 μg·kg^–1^), and DS3 (DEX 1.5 μg·kg^–1^). The Ramsay sedation scale was maintained at 3 in all groups. Results showed that anesthesia onset time was shorter, and the perioperative hemodynamic profile was more stable, in the DS3 group. The number of intraoperative movements was also lowest in the DS3 group. The time to first dose of rescue midazolam and lidocaine was significantly longer, but the total corresponding accumulated doses were lower in the DS3 group. Although the time to recovery prior to discharge from the post anesthesia care unit was longer, the overall incidence of tachycardia was lower in the DS3 group, and it received the highest bronchoscopist satisfaction score among the three groups. We therefore conclude that high-dose DEX plus SF can be safely and efficaciously used in children undergoing flexible bronchoscopy.

## INTRODUCTION

Flexible bronchoscopy has been widely used since first implemented in 1968 [1]. When performed in pediatric patients, general anesthesia and monitored anesthesia care (MAC) are preferred, as they provide higher success rates [2, 3]. However, flexible bronchoscopy represents a unique challenge for the anesthesiologist, as it requires maximizing both bronchoscopist and patient satisfaction [4]. Short acting opioids (fentanyl, remifentanil and sufentanil), newer drugs (dexmedetomidine), and modern ventilation technologies (supraglottic airways and mechanical jet ventilators) have facilitated the procedure. Nevertheless, medical teams are now opting for setting up a technical team with a dedicated anesthesiologist to deal with potentially increased adverse events [5–7].

Whereas general anesthesia is usually preferred for complex bronchoscopy, MAC (‘conscious sedation’ plus topical anesthesia) may be used for simple flexible bronchoscopy [8]. Benzodiazepines (midazolam), intravenous general anesthetics (propofol, etomidate, opioids), inhalational agents (sevoflurane, desflurane) or a combination of these drugs are commonly used during simple flexible bronchoscopy; however, each of these drugs has limitations [8, 9]. Benzodiazepines and intravenous general anesthetics, except opioids, have no analgesic properties. Opioids (fentanyl, sufentanil, remifentanil) can provide excellent analgesia but have modest sedative effects. Inhalational agents have unavoidable defects such as causing air pollution. Most important of all, inadequate combinations of these drugs can result in severe respiratory depression, which is the most common complication of flexible bronchoscopy [10–12]. Thus, the need to optimize drug combinations during this procedure is critical, especially when it is performed in children.

Dexmedetomidine (DEX), a highly selective α2 adrenergic receptor agonist, has a more favorable pharmacokinetic profile than clonidine [13]. Previous studies have reported that DEX, as compared with midazolam, propofol, fentanyl, and remifentanil, could be safely and effectively used for bronchoscopic procedures [14, 15]. However, according to a search for English language articles published between 1995 and 2015 on MEDLINE, PubMed, EMBASE, Cochrane Central Register of Controlled Trials, and Web of Science that included the terms dexmedetomidine, sufentanil, pediatric, children, and flexible bronchoscopy, did not yield any studies reporting on the safety and efficacy of DEX plus SF in children undergoing flexible bronchoscopy. To address this evidence gap, we conducted the present retrospective trial.

## RESULTS

### Baseline characteristics

**Figure 1:**
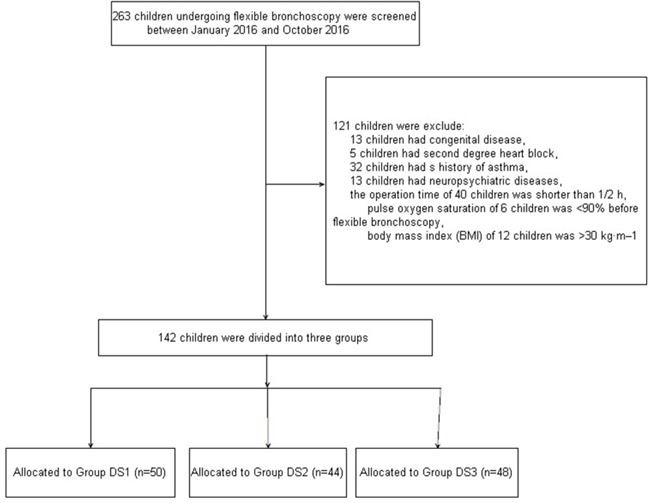
Patient enrollment flow diagram

263 children undergoing flexible bronchoscopy were screened between January 2016 and October 2016 (Figure 1). 121 children were excluded: 13 who had congenital diseases, 5 who had second degree heart block, 32 with history of asthma, 13 with neuropsychiatric diseases, 40 in whom the operation time was shorter than 1/2 h, 6 with a pulse oxygen saturation <90% prior to the procedure, and 12 with a BMI >30 kg·m^–2^. In total, 142 children were included in the study. They were divided into three groups (DS1, n = 50; DS2, n = 44; and DS3, n = 48). Demographic and baseline clinical parameters were not significantly different among the three groups (Table 1).

**Table 1:** Demographic and baseline clinical parameters

Variable	Group DS1(n=50)	Group DS2(n=44)	Group DS3(n=48)	*P*
Age (y)	9.54±2.84	10.07±2.46	9.34±2.74	0.412
Body weight (kg)	35.98±6.48	34.51±7.82	35.22±6.90	0.602
Sex (male/female)	37/13	30/14	35/13	0.805
BMI (kg·m^–2^)	27.15±2.31	27.48±3.05	26.88±3.26	0.611
ASA (I/II)	38/12	35/9	35/13	0.758
Duration of anesthesia (min)	40.23±11.76	43.15±12.03	39.74±10.54	0.311
Duration of bronchoscopy (min)	37.42±4.53	39.16±5.44	38.74±5.20	0.217
Type of bronchoscopy, n (%)				
Inspection	35 (70.00%)	33 (75.00%)	32 (66.67%)	
Bronchoalveolar lavage	8 (16.00%)	6 (13.64%)	8 (16.67%)	0.922
Transbronchial biopsy	2 (4.00%)	3 (6.82%)	4 (8.33%)	
Others	5 (10.00%)	2 (4.55%)	4 (8.33%)	

Variables presented as mean±SD or number of patients, n (%). BMI=body mass index; ASA=American Society of Anesthesiology.

### Intraoperative variables

**Figure 2:**
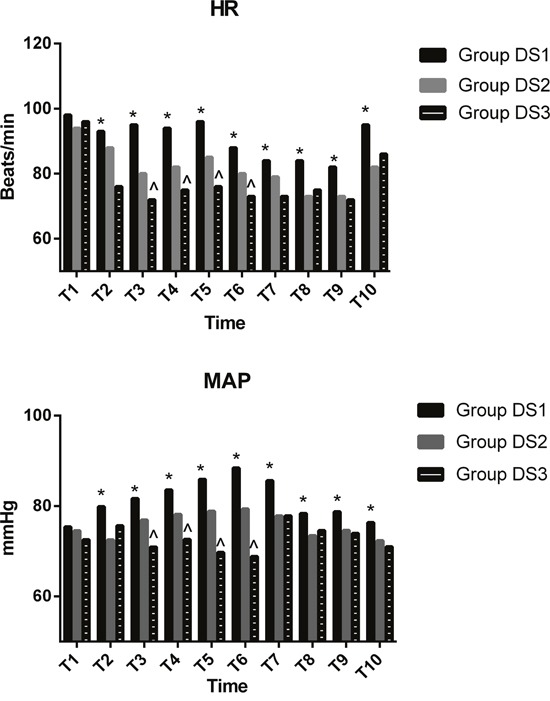
Hemodynamic measurements Baseline hemodynamic values were similar among the three groups. Compared with the DS1 group, both HR and MAP were significantly decreased from T2 to T10 in the DS2 and DS3 groups (*P* < 0.05). Compared with the DS2 group, both HR and MAP were significantly decreased from T3 to T7 in the DS3 group (*P* < 0.05). T1, arrival at the operating room; T2, after bolus administration of drug; T3, at the initiation of flexible bronchoscopy; T4, 5 min after initiation of bronchoscopy; T5, 10 min after initiation of bronchoscopy; T6, at the end of bronchoscopy; T7, 5 min after arriving at PACU; T8, 10 min after arriving at PACU; T9, 20 min after arriving at PACU; T10, 30 min after arriving at PACU; *P < 0.05 vs Group DS2 and Group DS3, ^ *P* < 0.05 vs Group DS2.

Baseline hemodynamic measurements were similar among the three groups (Figure 2). Compared with the DS1 group, both heart rate (HR) and mean arterial pressure (MAP) were significantly decreased from T2 to T10 in the DS2 and DS3 groups (*P <* 0.05, Figure 2). Compared with the DS2 group, both HR and MAP were significantly decreased from T3 to T6 in the DS3 group (*P <* 0.05, Figure 2).

**Figure 3:**
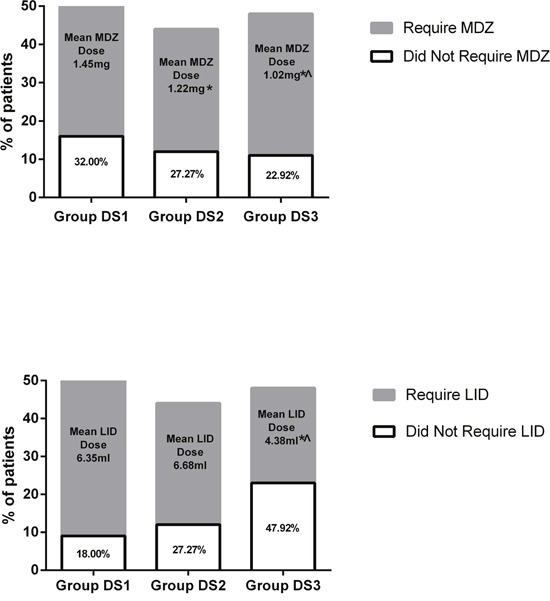
Midazolam and lidocaine use and dosage *P < 0.05 vs Group DS2 and Group DS3, ^ *P* < 0.05 vs Group DS2.

Among the three groups, anesthesia onset time was significantly shorter in DS3 (16.02 ± 3.49 *vs* 14.89 ± 4.23 *vs* 12.11 ± 3.44 min, *P* < 0.001, Table 2). Total dose of rescue midazolam was significantly higher in DS1 and DS2 than in DS3 (1.45 ± 0.47 *vs* 1.22 ± 0.43 *vs* 1.02 ± 0.34 mg *P* < 0.001, Table 2, Figure 3), while total dose of rescue lidocaine was significantly lower in DS3 (6.35 ± 2.09 *vs* 6.68 ± 2.11 *vs* 4.38 ± 1.25 ml, *P* < 0.001, Table 2, Figure 3). Times to first dose of rescue midazolam (9.36 ± 2.46 *vs* 11.82 ± 3.23 *vs* 13.80 ± 3.13 min, *P* < 0.001, Table 2) and lidocaine (10.43 ± 2.85 *vs* 12.41 ± 2.98 *vs* 14.52 ± 3.57 min, *P* < 0.001, Table 2) were significantly longer in DS3. Total cumulative dose of DEX was higher in DS3 (38.56 ± 10.98 *vs* 46.25 ± 16.42 *vs* 55.47 ± 14.22 μg, *P* < 0.001, Table 2). Total cumulative dose of SF was higher in DS1 (23.38 ± 4.02 *vs* 19.49 ± 4.11 *vs* 17.94 ± 3.87 μg, *P* < 0.001, Table 2).

**Table 2:** Intraoperative variables

Variable	Group DS1(n=50)	Group DS2(n=44)	Group DS3(n=48)	*P*
Anesthesia onset time (min)	16.02±3.49	14.89±4.23	12.11±3.44*^	0.000
Time to first dose of rescue midazolam (min)	9.36±2.46	11.82±3.23*	13.80±3.13*^	0.000
Time to first dose of rescue lidocaine (min)	10.43±2.85	12.41±2.98*	14.52±3.57*^	0.000
Total dose of rescue midazolam (mg)	1.45±0.47	1.22±0.43*	1.02±0.34*^	0.000
Total dose of rescue lidocaine (ml)	6.35±2.09	6.68±2.11	4.38±1.25*^	0.000
Total dose of dexmedetomidine (μg)	38.56±10.98	46.25±16.42*	55.47±14.22*^	0.000
Total dose of sufentanil (μg)	23.38±4.02	19.49±4.11*	17.94±3.87*	0.000
Total patient movements, n (%)	26 (52.00%)	18 (40.91%)	11 (22.92%)*	0.012
converted to propofol, n (%)	8 (16.00%)	6 (13.64%)	5(10.42%)	0.747

Variables presented as mean±SD or number of patients, n (%). *P < 0.05 vs Group DS1, ^*P* < 0.05 vs Group DS2.

The total number of movements during the bronchoscopy was lower in the DS3 group (52.00% *vs* 40.91%*vs* 22.92%, *P* = 0.012, Table 2). Eight children from DS1, 6 children from DS2, and 5 children from DS3 required an alternative sedative (propofol) to complete the procedure (*P* = 0.747, Table 2). There were significant differences among the three groups in terms of the overall incidence of tachycardia (46.00% *vs* 38.64% *vs* 25.00%, *P* = 0.042, Table 3). However, there was no difference among groups in vasoactive drug usage (Table 4).

**Table 3:** Adverse events

Variable	Group DS1(n=50)	Group DS2(n=44)	Group DS3 (n=48)	*P*
Tachycardia	23 (46.00%)	17 (38.64%)	12 (25.00%)*	0.042
Hypertension	20(40.00%)	15 (34.09%)	11 (22.92%)	0.188
Bradycardia	7 (14.00%)	10 (22.73%)	12 (25.00%)	0.362
Hypotension	3 (6.00%)	6 (13.64%)	5(10.42%)	0.468
Nausea	14 (28.00%)	12 (27.27%)	13 (27.08%)	0.994
Vomiting	4 (8.00%)	6 (13.64%)	5 (10.42%)	0.674
Cough	28 (56.00%)	22 (50.00%)	18 (37.50%)	0.176
Hypoxemia	16 (32.00%)	12 (27.27%)	8 (16.67%)	0.205

Variables presented as number of patients, n (%). *P < 0.05 vs Group DS1, ^*P* < 0.05 vs Group DS2.

**Table 4:** Vascular active drug usage during bronchoscopy

Variable	Group DS1(n=50)	Group DS2 (n=44)	Group DS3 (n=48)	*P*
Urapidil	16 (32.00%)	12 (27.27%)	8 (16.67%)	0.205
Esmolol	18 (36.00%)	13 (29.55%)	9 (18.75%)	0.166
Ephedrine	2 (4.00%)	5 (11.36%)	3 (6.25%)	0.384
Atropine	5 (10.00%)	8 (18.18%)	9 (18.75%)	0.399

Variables presented as number of patients, n (%).

### Postoperative variables

Recovery time before discharge from the post anesthesia care unit (PACU) (time to an Aldrete score ≥9) was significantly longer in the DS3 group compared with the other two groups (12.35 ± 3.48 *vs* 13.78 ± 3.82 *vs* 15.35 ± 4.02, *P* = 0.001, Table 5). Bronchoscopist satisfaction scores showed significant higher in DS3 than in DS1 and DS2 (*P =* 0.008, Table 5).

**Table 5:** Postoperative variables

Variable	Group DS1(n=50)	Group DS2(n=44)	Group DS3(n=48)	*P*
Recovery time (min)	12.35±3.48	13.78±3.82	15.35±4.02*^	0.001
Bronchoscopist satisfaction score	2.75 (1.75-3.25)	3.25 (2.25-3.75)*	3.75 (2.75-4.00)*^	0.008

Variables presented as mean±SD or median (interquartile range). *P < 0.05 vs Group DS1, ^*P* < 0.05 vs Group DS2.

## DISCUSSION

Our results show that flexible bronchoscopy under the high DEX, DS3 protocol (i.e. DEX infusion at 1.5 μg·kg^–1^ for 10 min, then adjusted to 0.5-0.7 μg·kg^–1^·h^–1^; SF infusion at 0.3 μg·kg^–1^ for 10 min, then adjusted to 0.1-0.3 μg·kg^–1^·h^–1^) shortened the anesthesia onset time, decreased the number of intraoperative patient movements and the overall incidence of tachycardia, and resulted in a more stable perioperative hemodynamic profile. Children in the DS3 group also needed a lower dose of rescue midazolam and lidocaine to complete the procedure, and the time to the first dose of rescue medication was also significantly longer in this group. For these children, however, the recovery time prior to discharge from the PACU was significantly longer than for those in the other two groups.

Rigid bronchoscopy is usually used for airway or esophageal foreign body removal in adults and children, while flexible bronchoscopy is usually used by respiratory physicians and pediatricians for the diagnosis and treatment of respiratory diseases [16, 17]. General anesthesia is still the gold standard technique for rigid bronchoscopy, and also for complex procedures where a flexible bronchoscope is used. In contrast, most flexible bronchoscopies can be done under topical anesthesia, MAC, or general anaesthesia [3]. MAC, i.e ‘conscious sedation’, has been recently used in many clinical fields, particularly in simple flexible bronchoscopy, as an alternative to topical anesthesia [18, 19]. Benzodiazepines have sedative, hypnotic, anxiolytic, anticonvulsant, and muscle relaxing effects and are one of the most commonly used sedatives during bronchoscopy. Because of its short elimination half-life and a fast onset of action, midazolam is a first-choice drug among the benzodiazepines. However, its pharmacological effects vary among patients, and it may depress the ventilatory drive and finally cause apnea, especially in patients with co-morbidities who are taking other respiratory depressant drugs [20, 21]. As a short-acting anesthetic agent with rapid recovery, propofol has been widely used in many clinical fields such as gastrointestinal endoscopy, thyroplasty, bronchoscopy, interventional or radiological procedures, and during awake bronchoscopy intubation, for its hypnotic effect [22, 23]. Ketamine, on the other hand, has been increasingly used for flexible bronchoscopy in children for its sympatholytic and analgesic effects, as well as for its potent bronchodilator actions. However, it increases salivation and mucous secretions, and upper airway reflexes are preserved [24]. Though opioids are frequently used during bronchoscopy for its analgesic properties, they may lead to bradycardia, hypotension, and hypoxemia when in high doses or combined with other sedative drugs.

A usual solution for these problems, adopted recently by bronchoscopists and anesthesiologist, has been combining two or more agents [14, 25]. As a mu opioid receptor, SF has short half-life and its analgesic potency is 5-10 times that of fentanyl. Previous studies have in fact reported the use of remifentanil in combination with propofol for flexible bronchoscopy in children [11]. DEX, a new selective α2-agonist, has sedative, anxiolytic and analgesic effects. Furthermore, it has the advantage of causing mild respiratory depression, even at higher doses. Previous studies have reported that DEX can both decrease the incidence of desaturation and reduce tracheobronchial secretions [26]. A general recommendation is a DEX bolus infusion of 1 μg·kg^–1^ for 10 min, followed by maintenance infusion at a rate of 0.2-0.7 μg·kg^–1^·h^–1^, and SF infusion at 0.3 μg·kg^–1^ for 10 min, then adjusted to 0.1-0.3 μg·kg^–1^·h^–1^ for maintenance. However, as DEX has a larger apparent volume of distribution in children than in adults, children may need larger initial doses of DEX to reach comparable steady-state plasma levels, although the maintenance doses are similar [27]. Therefore, in this trial we adopted DEX infusion at 0.5-1.5 μg·kg^–1^ for 10 min, then adjusted it to 0.5-0.7 μg·kg^–1^·h^–1^. Non-autonomous movements are one of the most common reasons of failure of flexible bronchoscopy in children. However, only a small number of children required conversion to propofol, due perhaps to the complementary drug effects in these studies.

Comparisons among the three groups revealed that anesthesia onset time was significantly shorter in the DS3 group. At the same time, fewer children in this group needed rescue drugs to complete the bronchoscopy, presumably as a result of better hemodynamic stability and the synergic sedative effects of DEX and SF at high doses. Though bradycardia and hypertension are the most common adverse reactions reported during bolus infusion of DEX, we did not observe differences in their incidence among the three groups, partly because they were counteracted by premedication in our study [11]. There were however significant differences among groups in the overall incidence of tachycardia (the lowest one observed in DS3) although vasoactive drug use was similar. The reasons for this may be ascribed to the different DEX and SF dosages used in the three groups. The overall time to recovery before discharge from the PACU was significantly shorter than reported previously [28]. However, compared with DS1 and DS2, recovery took longer for children in the DS3 group. On the other hand, bronchoscopist satisfaction scores were significantly higher in DS3, which may be due to the fewer intraoperative patient movements recorded for this group.

In summary, our results show that high dose of DEX-SF (DEX infusion at 1.5 μg·kg^–1^ for 10 min, then adjusted to 0.5-0.7 μg·kg^–1^·h^–1^; SF infusion at 0.3 μg·kg^–1^ for 10 min, then adjusted to 0.1-0.3 μg·kg^–1^·h^–1^), provided better and stable hemodynamic profiles, lower use of rescue medication, fewer intraoperative movements, and a higher bronchoscopist satisfaction score, although the recovery time was longer. We conclude that this sedation protocol could be safely and effectively used in children undergoing flexible bronchoscopy.

There are several limitations in this study. First, as it is a retrospective trial in a single medical center, a multicenter prospective controlled trial would be necessary to verify the superiority of high dosage DEX-SF in children undergoing flexible bronchoscopy. Second, due to technical limitations we did not measure patients’ serum concentrations of DEX and SF. Third, we did not collect blood gas measurements or performed transcutaneous capnography, which may be more accurate to assess the respiratory state of our patients [28–30]. Finally, as we only discussed three different combinations of DEX-SF, studies should be carried out to verify the efficacy of different doses of DEX-SF for pediatric flexible bronchoscopy.

## MATERIALS AND METHODS

### Patients

Approval for this retrospective clinical trial was obtained from the Institutional Review Board of Qilu Children's Hospital of Shandong University. Children undergoing flexible bronchoscopy between January 2016 and October 2016 were enrolled in this study after obtaining written informed consent of their parents if they met the following inclusion criteria: age between 7 and 12 years old, and American Society of Anesthesiology (ASA) grade I to II. Exclusion criteria included congenital disease, neuropsychiatric diseases, second or third degree heart block, asthma, operation time shorter than 1/2 h, pulse oxygen saturation <90% before flexible bronchoscopy, and body mass index (BMI) >30 kg/m^2^.

Children were divided into three groups: DS1 (n = 50, DEX infusion at 0.5 μg·kg^–1^ for 10 min, then adjusted to 0.5-0.7 μg·kg^–1^·h^–1^; SF infusion at 0.3 μg·kg^–1^ for 10 min, then adjusted to 0.1-0.3 μg·kg^–1^·h^–1^), DS2 (n = 44, DEX infusion at 1 μg·kg^–1^ for 10 min, then adjusted to 0.5-0.7 μg·kg^–1^·h^–1^; SF infusion at 0.3 μg·kg^–1^ for 10 min, then adjusted to 0.1-0.3 μg·kg^–1^·h^–1^), and DS3 (n = 48, DEX infusion at 1.5 μg·kg^–1^ for 10 min, then adjusted to 0.5-0.7 μg·kg^–1^·h^–1^; SF infusion at 0.3 μg·kg^–1^ for 10 min, then adjusted to 0.1-0.3 μg·kg^–1^·h^–1^). All children and their parents were explained about the operative procedure on the day before surgery. Flexible bronchoscopy was performed by the same bronchoscopist, who has completed ten years of residency.

Children were fasted for 6 hours for solids and for 2 hours for clear fluids before the procedure [31]. After baseline hemodynamics were recorded, midazolam 0.03 mg·kg^–1^ and atropine 0.01mg·kg^–1^ were given intravenously in the operating room [8]. Five-lead electrocardiography, noninvasive arterial blood pressure, peripheral pulse-oximetry (SpO_2_), respiratory rate (RR), and temperature (TEM) were continuously monitored using an automated system (Philips IntelliVue MP70). A forced-air warming device (EQUATOR Convective Warmer, EQ-5000) was used during the procedure to maintain normothermia.

### Flexible bronchoscopy

After children received oxygen supplementation at 4 L·min^–1^ through a nasal cannula, loading doses of both DEX and SF were infused for 10 min, followed by continuous drug maintenance infusion; topical anesthesia was then performed using 2 ml of 1% lidocaine spray applied twice into the oral cavity. Upon visualization of the vocal cords, trachea, and the right and left main bronchi, 4 ml of 1% lidocaine were delivered through the flexible bronchoscope channel to suppress the cough reflex. Once the Ramsay sedation score reached 3 (children still responding to commands), flexible bronchoscopy was performed [32]. Whenever indications of insufficient sedation were observed during the procedure, a rescue bolus of midazolam 0.03 mg·kg^–1^ was given, repeated every 5 min to a maximum dose of 1.5 mg. Whenever indications of inadequate analgesia were observed during the procedure, an additional 2 ml of 1% lidocaine was administered through the side hole of the flexible bronchoscope to a maximum dose of 10 ml. The amount of midazolam and lidocaine administered was recorded. If the patient did not reach an optimal sedation status after the maximum dose of midazolam and lidocaine, propofol 1.5 mg·kg^–1^ was provided. DEX and SF infusion were stopped 5 min before the end of the procedure, after which all children received tropisetron 0.1 mg·kg^–1^ before being transferred to the PACU.

On arrival at the PACU, children were continuously monitored by five-lead electrocardiography, noninvasive arterial blood pressure and SpO_2_ for at least 12h. Hemodynamics [HR, noninvasive blood pressure, SpO_2_, TEM] were monitored every 5 min for the first 20 min, then every 10 min until children were discharged (Aldrete Score ≥9) [33]. Bronchoscopist satisfaction was assessed 24h after bronchoscopy [34].

### Adverse events

Bradycardia and tachycardia were defined as a ≥30% reduction or increase of baseline HR, and treated with intravenous atropine 0.01 mg·kg^–1^ or esmolol 0.3 mg·kg^–1^, respectively. Hypertension and hypotension were defined as a ≥30% increase or decrease from baseline mean arterial blood pressure, and treated with intravenous urapidil 0.02 mg·kg^–1^ or ephedrine 0.01 mg·kg^–1^, respectively. Hypoxemia was defined as an SpO_2_ <90% for >30s, and treated with oxygen supplementation at 6 L·min^–1^ or with verbal and tactile stimulation, chin lifts, jaw thrusts, a face mask, and manual ventilation [35].

### Outcome variables

Intraoperative hemodynamic parameters (HR, noninvasive blood pressure, SpO_2_, TEM) were recorded at the following time points: upon arrival at the operating room (T1), after bolus administration of drugs (T2), at the initiation of flexible bronchoscopy (T3), 5 min after initiation of bronchoscopy (T4), 10 min after initiation of bronchoscopy (T5), at the end of bronchoscopy (T6) and 5 min (T7), 10 min (T8), 20 min (T9), and 30 min (T10) after being transferred to the PACU. Anesthesia onset time, the number of intraoperative movements, total cumulative doses of dexmedetomidine, sufentanil, midazolam, and lidocaine, time to first dose of rescue midazolam and lidocaine, postoperative recovery time, adverse events, and bronchoscopist satisfaction score were also recorded.

### Statistical analysis

The Kolmogorov–Smirnov test was used to assess the distribution of variables. Homogeneity of variances was determined using Levene's tests. Quantitative data was expressed as mean and standard deviation or median and inter-quartile range (IQR). Inter-group comparisons were performed using repeated-measures analysis of variance. The Bonferroni's correction was used for post-hoc multiple comparisons. The nonparametric Kruskal-Wallis test was used for variables that were not normally distributed. Categorical data was expressed as frequency and percentage and analyzed using chi-squared tests or Fisher's exact tests when appropriate. *P* <0.05 was considered statistically significant. Statistical analysis was performed with SPSS for Windows version 18.0 (SPSS Inc. Chicago, IL, USA).

## Abbreviations

DEX: dexmedetomidine
SF: sufentanil
MAC: monitored anesthesia care
HR: heart rate
MAP: mean arterial pressure
PACU: post anesthesia care unit
BMI: body mass index
ASA: American Society of Anesthesiology
SpO_2_: pulse-oximetry
RR: respiratory rate
TEM: temperature
IQR: inter-quartile range
